# Three-dimensional reconstruction of brain structures of the rodent *Octodon degus*: a brain atlas constructed by combining histological and magnetic resonance images

**DOI:** 10.1007/s00221-013-3667-1

**Published:** 2013-08-31

**Authors:** Noriko Kumazawa-Manita, Mariko Katayama, Tsutomu Hashikawa, Atsushi Iriki

**Affiliations:** Laboratory for Symbolic Cognitive Development, RIKEN, Brain Science Institute, Wako, Saitama 351-0198 Japan

**Keywords:** Brain atlas, Rodent, Degu, Three-dimensional reconstruction, Magnetic resonance imaging, Nissl staining

## Abstract

Degus (*Octodon degus*) are rodents that are becoming more widely used in the neuroscience field. Degus display several more complex behaviors than rats and mice, including complicated social behaviors, vocal communications, and tool usage with superb manual dexterity. However, relatively little information is known about the anatomy of degu brains. Therefore, for these complex behaviors to be correlated with specific brain regions, a contemporary atlas of the degu brain is required. This manuscript describes the construction of a three-dimensional (3D) volume rendered model of the degu brain that combines histological and magnetic resonance images. This atlas provides several advantages, including the ability to visualize the surface of the brain from any angle. The atlas also permits virtual cutting of brain sections in any plane and provides stereotaxic coordinates for all sections, to be beneficial for both experimental surgeries and radiological studies. The reconstructed 3D atlas is freely available online at: http://brainatlas.brain.riken.jp/degu/modules/xoonips/listitem.php?index_id=24.

**Electronic supplementary material:**

The online version of this article (doi:10.1007/s00221-013-3667-1) contains supplementary material, which is available to authorized users.

## Introduction

The caviomorph rodent *Octodon degus*, commonly called the trumpet-tailed rat or the degu, is widely used as an animal model of human diseases. Over the last several decades, degus were used to study diabetes, hyperglycemia, pancreatic function, and adaptation to high altitude (Wright and Kern 1992). In addition, degus are also becoming more widely used in the neuroscience and neurological medicine fields (Braidy et al. 2012). Recent studies used degus to assess the relationship between sociality and cognitive brain functions (Helmeke et al. 2009), and the evolutionary aspects of the acquisition of tool-use abilities (Okanoya et al. 2008). Furthermore, degus are biparental animals. Paternal care of degus regulates the developmental expression pattern of CRF-expressing interneurons in the amygdala and hippocampus (Seidel et al. 2011) and the development of catecholaminergic innervation of the prefrontal cortex and related limbic brain regions (Braun et al. 2012). Thus, degus have unique physical and behavioral features that are beneficial for various developmental and evolutionary studies. Degus are often a more suitable research model than rats or mice because of these unique characteristics.

Studies using degus have uncovered several interesting brain functions and dysfunctions associated with specific behaviors. However, these behaviors are generally associated with broad brain regions (Ardiles et al. 2012; Bock et al. 2012; Braun et al. 2012). Degu studies have also led to the discovery of neuropathological loci that may be relevant for Alzheimer’s disease (Braidy et al. 2012). However, these studies also lacked precise localization of defects to specific brain structures. These difficulties are due in part to a lack of appropriate maps that precisely define brain structures in degus.

Precise maps of brain structures also permit the application of newer technologies to analyze brain functions in animal models. Positron emission tomography (Virdee et al. 2012) and magnetic resonance imaging (MRI) (Higuchi et al. 2005) techniques are commonly used to examine brain metabolism in small animals such as mice. However, the lack of precise maps of degu brain structures severely limits the usefulness of these technologies for degu-based research.

To precisely assess degu brain structures in behavioral, histological, and imaging studies, a contemporary degu brain atlas is required. Several rodent brain atlases are currently used (Paxinos et al. 1980; Purger et al. 2009), including one of the degu brain (Wright and Kern 1992). However, current studies that utilize techniques such as magnetic resonance (MR) images require histologically detailed brain atlases. There are no high-resolution degu brain atlases currently available. This study describes the construction of a Web-accessible, three-dimensional (3D), digital volume rendered degu brain atlas. This degu brain atlas was constructed by combining MR images with full-color histological images. The atlas can be freely rotated and allows virtual sectioning and axis readjustments. These parameters provide practical guidance for stereotaxic surgeries and electrophysiological experiments in addition to a map of annotated brain structures.

The main purpose of this study was to provide a structural guide that can be used to map brain functions in degus. Degus show human-like social and skilled behaviors that make them a more appropriate model for some studies than mice and rats. For these reasons, degus are becoming an essential experimental animal model in the study of human neurological diseases. However, current degu-based research lacks information about precise brain regions. Findings are usually correlated with broad brain regions such as the cerebral cortex. Thus, the precise degu brain atlas presented in this study will permit more in depth and higher quality studies.

## Materials and methods

The brain atlas was based on analyses of six degu brains. These degus weighed 218.0–258.1 g (mean ± SEM: 235.9 ± 6.8) and were obtained from colonies maintained in the Laboratory for Symbolic Cognitive Development, BSI, RIKEN. The degus were housed under environmentally enriched conditions in steel cages (width × length × height: 90 × 45 × 45 cm) with their families. Each cage contained running wheels, a wood house, and a wood tunnel. The housing room had a 12-h light/dark cycle with ambient temperatures (near 20 °C). The degus had ad libitum access to food and fresh water. The drinking water contained vitamin C supplements because degus are unable to generate it. The horizontal and coronal zero axes were adjusted when the degus were in a stereotaxic apparatus to the line through the maxillary incisor and external acoustic meatus and the interaural line, respectively. The distances between coronal zero and the suture bregma were calculated for each degu and used as brain size variation indices. These distances ranged from 5.9 to 7.2 mm (mean ± SEM: 6.8 ± 0.2). All experimental protocols were approved by the RIKEN Animal Experiment Committee and were conducted in accordance with the US National Institute of Health Guide for the Care and Use of Laboratory Animals.

Each degu was deeply anesthetized with sodium pentobarbital (200 mg/kg delivered by intraperitoneal injection), sequentially perfused through the left ventricle with 20 ml of 0.8 % sodium chloride and 200 ml of 4 % paraformaldehyde (PFA) in 0.1 M phosphate buffer (pH 7.4) and then placed into a stereotaxic apparatus for small animals (Narishige, Tokyo, Japan). The incisor bar was elevated 4.0 mm above the horizontal plane of the ear bars so that the horizontal zero plane passed through the anterior commissure and posterior commissure (AC–PC) line (radiological coordinates) (Wright and Kern 1992). Two reference tracts were made perpendicularly to the horizontal plane through the surface of the cerebral cortex with carbon shafts (diameter = 0.2 mm). The first reference shaft was placed along the vertical plane +1.0 mm and 2.0 mm lateral to the midline in the right hemisphere and the second reference shaft was placed 5.0 mm anterior to the first reference shaft. An additional reference shaft was placed in the horizontal plane through the cerebellum 1.5 mm lateral to the midline and 0.7 mm above the horizontal zero. The degu heads were then removed from the bodies and were stored for approximately 2 days in 4 % PFA at 4 °C. The lower jaws and soft tissues around the skulls were then removed, and the skulls with the intact brains were transferred to MRI holders. MRI data were acquired and then the skulls were placed in stereotaxic head holders with the interaural-incisor lines defined as horizontal zero (neurosurgical coordinates). The brain surfaces were then exposed. Pictures were taken to be used as alignment guides and then the brains were removed from the skulls and prepared for histological examinations. The brains were immersed in 30 % sucrose until they sunk and were then immersed in 10 % gelatin at 60 °C for 1 h. The brains were then embedded in 10 % gelatin at 4 °C. The gelatin-embedded brains were immersed in 4 % PFA for a few days and were then immersed again in 30 % sucrose until they sunk. Serial coronal sections were cut at a thickness of 50 μm on a freezing microtome. The section planes were then referred to the pictures taken when the skulls were in the stereotaxic head holder and were adjusted to the neurosurgical stereotaxic coordinates. The sections were then Nissl-stained with thionin to observe neurons. The radiological coronal plane was inclined at a 40 degree angle in the anterior direction when these sections were cut. This permitted coronal sections to be easily and reliably obtained by routine methods and removed the requirement of the sections to be adjusted to radiological coordinates.

Based on the calculations of distance between reference markers which made at surgery, this method of section preparation did not result in prominent shrinkage. Shrinkage effects were likely avoided by the gentle fixation of the brain in the skull. If partial shrinkage or wrinkles were observed in sections, then Nissl-stained images were replaced by images from neighboring sections to avoid errors in the image rendering.

The newly developed “SG-eye” software was used for 3D volume rendering of two-dimensional (2D) data (Fiatlux, Tokyo, Japan). SG-eye constructs 3D models by surface rendering and GPU accelerated volume ray-casting. The volume ray-casting algorithm consists of four steps: ray-casting, sampling, shading, and compositing. In the ray-casting step, a ray of sight is cast through the entire volume for each pixel. SG-eye contains several additional functions including position-adjusting, editing of mask images, and labeling and annotating brain regions. The SG-eye operating guide is explained in Fig. 5.

Photomicrographs in tif file formats were obtained from Nissl-stained sections with a NanoZoomer 2.0-HT digital slide scanner (Hamamatsu Photonics, Hamamatsu, Japan). The resolutions of the original tif images were decreased by 30 % so that the file sizes did not exceed 2 GB. Mask images of each brain region were prepared by subjecting Nissl-stained images to a path function in Photoshop (Adobe, San Jose, USA). The images were then imported into the SG-eye software to construct a 3D digital volume rendered brain model.

Magnetic resonance imaging holders with degu brains were also transferred to an MRI machine. MRI scans were performed using a 9.4-T Advance 400WB NMR spectrometer (Bruker Biospin GmbH: Rheinstetten, Germany) with a standard Micro 2.5 microimaging system. The inner diameter of the integrated transmitting and receiving coil was 25 mm. Each scan took approximately 1.5 h. The MR images adjusted to the Nissl section planes were used to construct 3D models with SG-eye software.

**Fig. 1:**
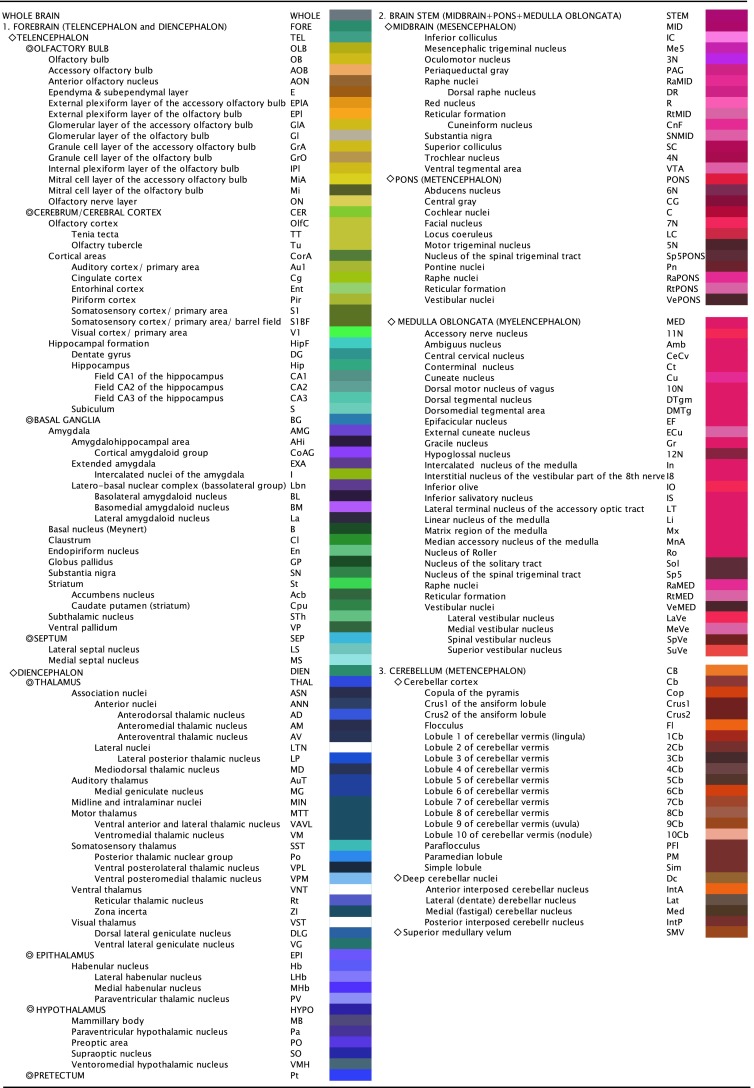
The annotated brain structures in the degu brain atlas are listed with their *abbreviations* and are arranged in a hierarchical organization (part 1 of 2). Each brain region was assigned a *unique color*. Users of the brain atlas can also search for structures by using the abbreviations

**Fig. 2:**
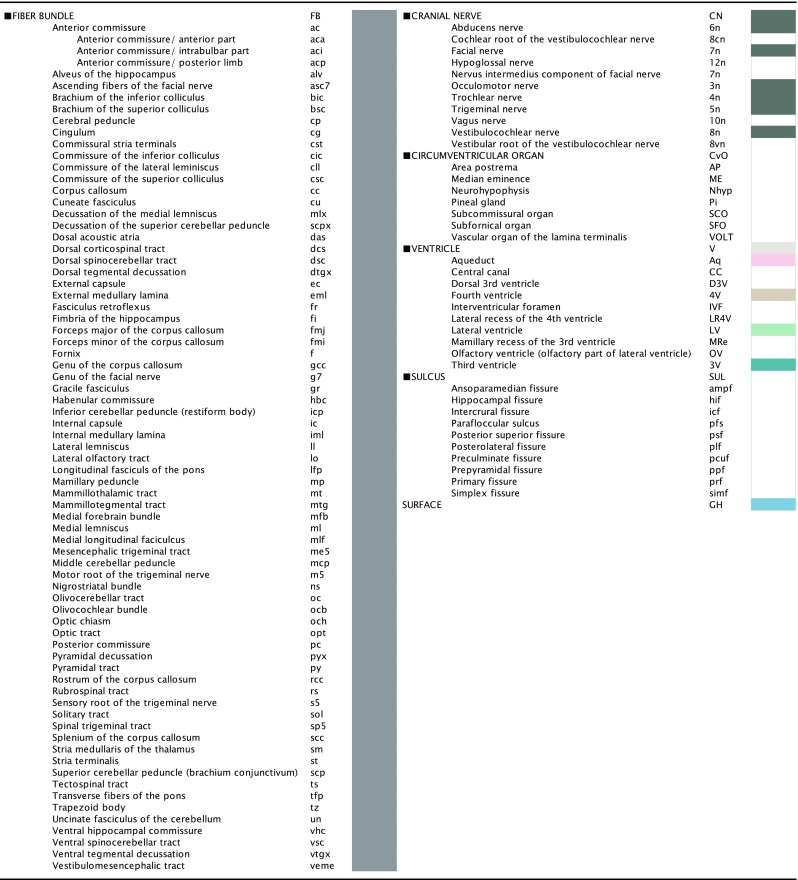
The annotated brain structures in the degu brain atlas are listed with their *abbreviations* and are arranged in a hierarchical organization (part 2 of 2). Each brain region was assigned a *unique color*. Users of the brain atlas can also search for structures by using the *abbreviations*

**Fig. 3:**
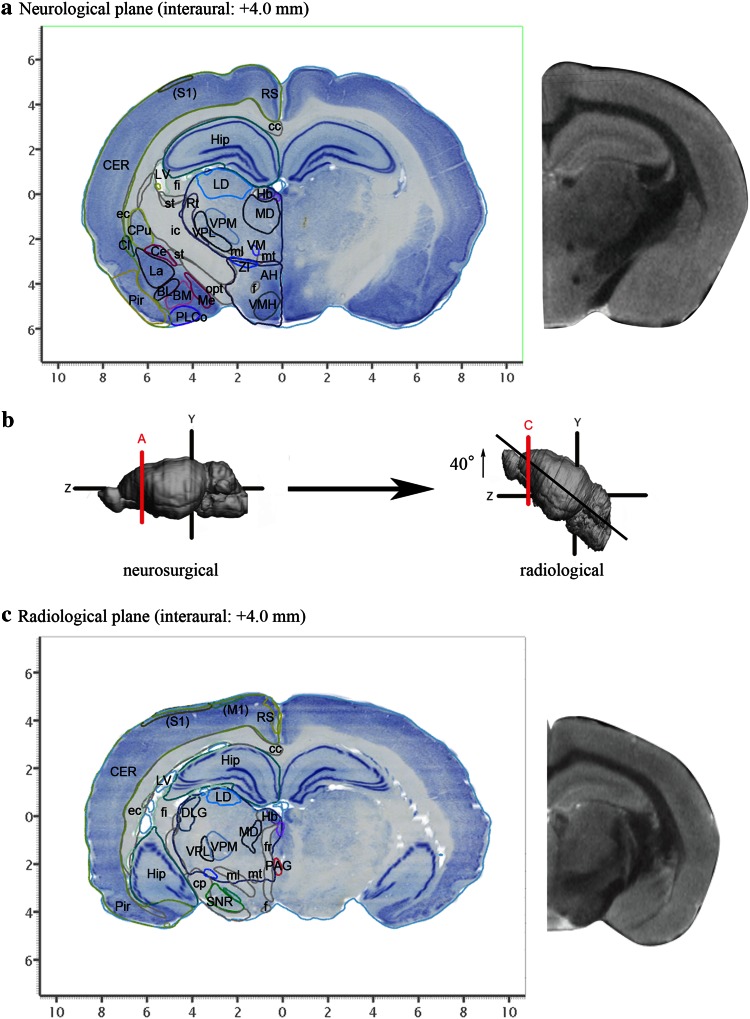
Representative sections reconstructed from the volume rendered brain model of the degu with neurosurgical (**a**) and radiological (**c**) stereotaxic coordinates. Nissl images with annotations are provided in the *left portion of each* *panel* and MR images taken from the corresponding levels are provided in the *right portion* of *each panel*. Locations with respect to the interaural line are indicated in *parenthesis* of each Nissl image. **b** The positional differences between neurosurgical (*left*) and radiological (*right*) stereotaxic coordinates. The radiological coordinates contain a horizontal zero axis that passes through the AC–PC line (Wright and Kern 1992). This axis results in an anterior shift of 40 degrees in radiological coordinates relative to neurosurgical coordinates. *Lines A* and *C* in **b** indicate approximate level of sectioning of Nissl images in **a** and **c**, respectively. *AH* anterior hypothalamic area, *BL* basolateral amygdaloid nucleus, *BM* basomedial amygdaloid nucleus, *cc* corpus callosum, *Ce* central amygdaloid nucleus, *CER* cerebral cortex, *Cl* claustrum, *cp* cerebral peduncle, *CPu* caudate putamen, *DLG* dorsal lateral geniculate nucleus, *ec* external capsule, *f* fornix, *fi* fimbria, *fr* fasciculus retroflexus, *Hb* habenular nuclei, *Hip* hippocampal formation, *ic* internal capsule, *La* lateral amygdaloid nucleus, *LD* laterodorsal thalamic nucleus, *LV* lateral ventricle, *(M1)* presumable primary motor cortex, *MD* mediodorsal thalamic nucleus, *Me* medial amygdaloid nucleus, *ml* medial lemniscus, *mt* mammillothalamic tract, *opt* optic tract, *PAG* periaqueductal gray, *Pir* piriform cortex, *PLCo* posterolateral cortical amygdaloid nucleus, *RS* retrosplenial cortex, *Rt* reticular thalamic nucleus, *(S1)* presumable primary somatosensory cortex, *SNR* substantia nigra, reticular part, *st* stria terminalis, *st* stria terminalis, *VM* ventromedial thalamic nucleus, *VMH* ventromedial hypothalamic nucleus, *VPL* ventral posterolateral thalamic nucleus, *VPM* ventral posteromedial thalamic nucleus, *ZI* zona incerta

**Fig. 4:**
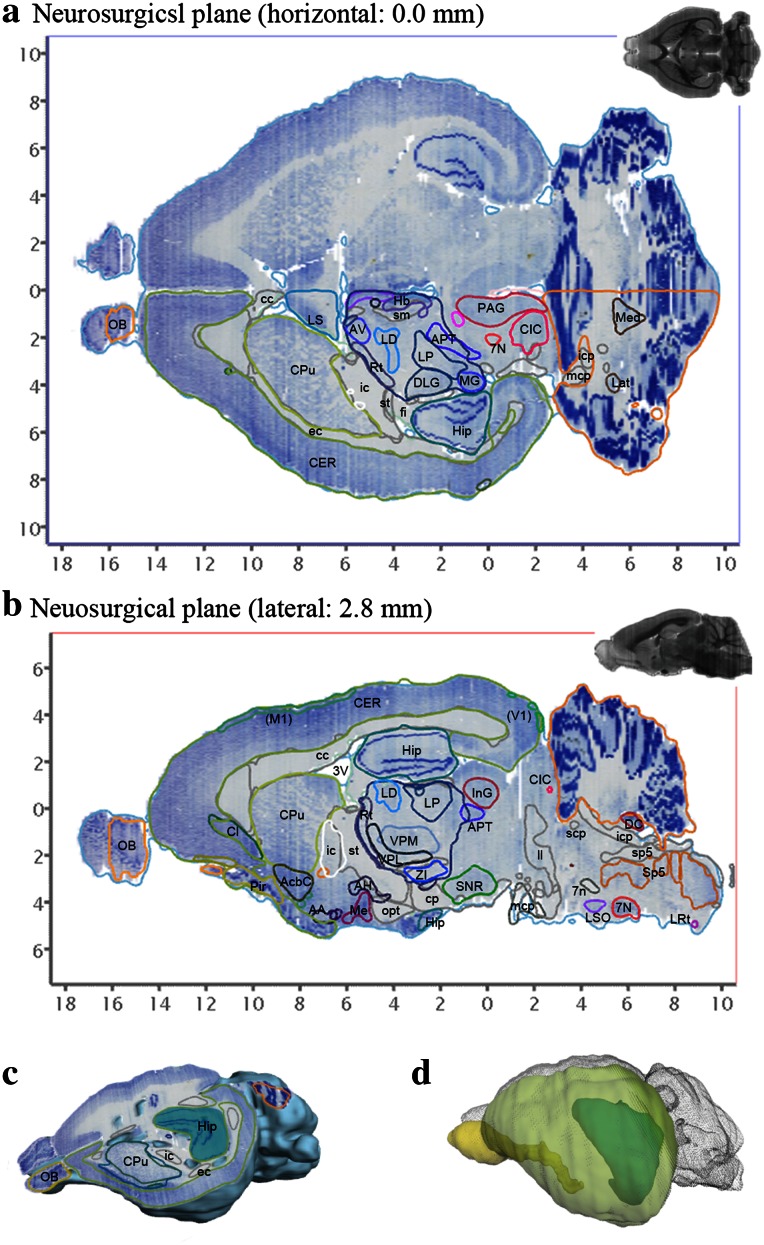
Representative examples of arbitrarily cut virtual sections and brain surface view using the 3D atlas. Brain sections can be virtually cut in any plane. The resulting sections display annotated Nissl images with outlines of brain regions and corresponding MR images. **a**, **b**, and **c** A horizontal, sagittal, and obliquely cut sections, respectively. **d** A combined view of surface of the cerebral cortex (labeled by *yellow green*) and some internal structures (olfactory bulb by *yellow* and hippocampus by *green/blue*). *3V* third ventricle, *7N* facial nucleus, *7n* facial nerve, *AA* anterior amygdaloid area, *AcbC* accumbens nucleus, core, *AH* anterior hypothalamic area, *APT* anterior pretectal nucleus, *AV* anteroventral thalamic nucleus, *cc* corpus calosum, *CER* cerebral cortex, *CIC* central nucleus of the inferior colliculus, *Cl* claustrum, *CPu* caudate putamen, *DC* dorsal cochlear nucleus, *DLG* dorsal lateral geniculate nucleus, *fmi* forceps minor, *Hb* habenular nuclei, *Hip* Hippocampal formation, *ic* internal capsule, *icp* inferior cerebellar peduncle, *InG* intermediate gray layer of the superior colliculus, *Lat* lateral cerebellar nucleus, *LD* laterodorsal thalamic nucleus, *ll* lateral lemniscus, *LP* lateral posterior thalamic nucleus, *LRt* lateral reticular nucleus, *LS* lateral septal nucleus, *LSO* lateral superior olive, *mcp* medial cerebellar peduncle, *Med* medial cerebellar nucleus, *Me* medial amygdaloid nucleus, *MGV* medial geniculate nucleus, ventral part, *OB* olfactory bulb, *opt* optic tract, *PAG* periaqueductal gray, *VPL* ventral posterolateral thalamic nucleus, *VPM* ventral posteromedial thalamic nucleus, *Rt* reticular thalamic nucleus, *scp* superior cerebellar peduncle, *sm* stria medullaris of the thalamus, *SNC* substantia nigra, compact part, *Sp5* nucleus of the spinal trigeminal tract, *sp5* spinal trigeminal tract, *ZI* zona incerta

The brain structures were assumed to be in the correct positions because the MR images were acquired from brains that were fixed in the skull. Finer data about the brain structures were then obtained from the Nissl-stained images because the resolutions of the Nissl-stained images (5,214 × 5,673, 72 pixels/inch) were much higher than the resolutions of the MR images (512 × 512, 72 pixels/inch). Figures 3 and 4 show representative Nissl-stained images and corresponding MR images obtained from a 22-month-old male degu (body weight: 245.0 g). In addition, the atlas also contains outlines with annotation of representative brain structures. The brain structures were delineated by referring to the published rodent (Jones 1985; Paxinos 2004), carnivores (Berman 1968; Berman and Jones 1982; Jones 1985), and primate histological descriptions (Jones 1985; Bloom et al. 1997, 1998, 1999). The fiber tracts were outlined by solid gray lines, and nuclei and cell groups were outlined by solid lines with unique colors assigned to each structure (Figs. 1, 2). Abbreviations were usually placed in the center of the structures. When this was not possible, the abbreviations were placed adjacent to the structures and the structures were indicated by lines. The outlines of the ventricles and aqueducts were made with solid colors. The previously published degu brain used Latin anatomical terms for the nomenclature (Wright and Kern 1992). By contrast, this atlas uses the English anatomical terms adopted in “The Rat Brain in Stereotaxic Coordinates” (Paxinos and Watson 2009) because this terminology is more widely used.

## Results

The volume rendered model was constructed from histological images obtained from Nissl-stained sections that were aligned with MR images. This method permitted the Nissl-stained images to be merged with the MR images and for the 3D brain atlas to be constructed from both types of information. The 3D reconstructed atlas is available at: http://brainatlas.brain.riken.jp/degu/modules/xoonips/listitem.php?index_id=24.

The hierarchical brain structures are listed in Figs. 1 and 2. Each major brain structure is labeled with an appropriate abbreviation and a unique color. Although annotated structures are small in number in the Figs. 3 and 4, other structures can also be identified in the digital atlas. Figure 3a shows coronal images with the neurosurgical stereotaxic coordinates that are commonly used for rodent brains. These coordinates were determined with a horizontal zero axis through the external acoustic meatus and maxillary incisor. In the Nissl sections, several representative structures are annotated.

Radiological stereotaxic coordinates can be reconstructed from this volume rendered model. As shown in Fig. 3b, the neurosurgical horizontal zero axis is inclined 40 degrees in the anterior direction relative to the radiological AC–PC line. Figure 3c shows annotated brain structures in coronal sections with converted radiological stereotaxic coordinates. Thus, this atlas can be used to identify structures in MR images taken with either neurosurgical or radiological coordinates.

There are several different useful applications of this degu brain atlas. In addition to the reconstruction of coordinates, this atlas can also be used to assess brain structures in sections that are cut in any plane. Brain structures in coronal, horizontal, and sagittal sections can all be determined from this atlas because all of the main structures are annotated in the volume rendered model (Figs. 1, 2). This application is particularly useful for in vitro electrophysiological experiments. If an experimenter wants to simultaneously record from several structures in a slice, then he/she can determine the plane of the slice by virtually simulating the sectioning with this atlas. While Agmon and Connors attempted to prepare slices in this manner with a tissue preparation, computer simulations with this atlas can be used to confirm the location of structures in the sections (Agmon and Connors 1991).

Another useful application of this atlas is that the entire brain surface can be reconstructed in 3D space by itself or with labeled structures of interest. This permits structural orientations to be easily visualized in the brain in 3D (Fig. 4). The surface images can be freely rotated. This function conveniently enables simple and rough localizations of brain areas that are being assessed.

**Fig. 5:**
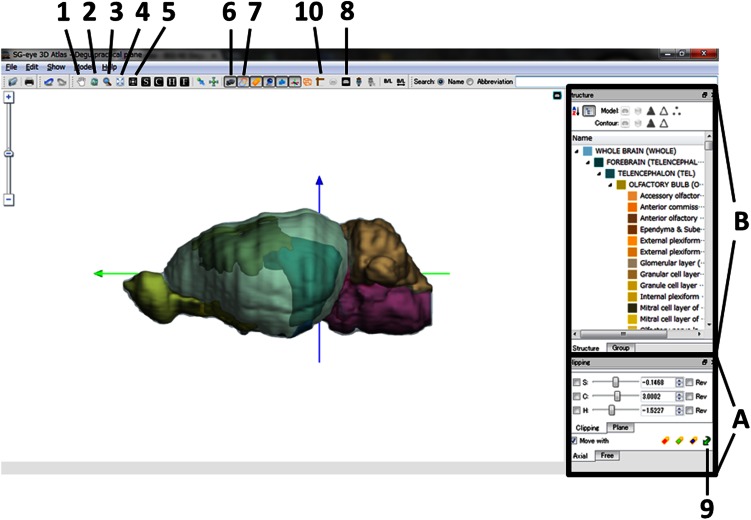
A guide to using the “SG-eye 3D Atlas” software. The *active buttons* in the *menu bar* are labeled (1–10). The functions of these *buttons* are explained in the Electronic Supplementary material and can be visualized in the tooltip window. Coronal sections can be visualized by manipulating the *bottom right* *corner panel* (labeled *A*). Specifically, the boxes labeled “C” and “Move with” in the “Clipping” and “Plane” modes in the “Axial” tab must be clicked. The “C” slider can be moved to change levels along the rostro-caudal axis. The *numbers* indicated in the *boxes* to the *right* of the sliders are the distance in mm from the interaural zero line. These *numbers* are either positive or negative to signify the rostral and caudal positions relative to the zero line, respectively. Sagittal and horizontal sections can be obtained in a similar fashion (click the “S” box for sagittal, or the “H” box for horizontal). 3D image or outlines can be removed or added by clicking the appropriate *buttons* (labeled 6 and 7). MR images can also be visualized by clicking the appropriate *button* (labeled 8). Coordinate transformations can be performed in the “Free” tab in the *bottom right control panel* (labeled *A*). These transformations can be performed on any combination of Nissl sections, MRI planes, and outlines by clicking the appropriate *buttons* (labeled *2*, *6*, *7*, and *8*) and for any plane of sections by clicking the appropriate buttons (the C, S, and H *buttons*). The hierarchical organization of the brain structures is indicated in the *middle right panel* (labeled *B*). Representative structures are indicated with unique *colors* in the image

There are also several features of the degu brain atlas that are useful for neuroanatomical or electrophysiological experiments. Arbitrarily sectioned planes and freely moving 3D stereograms can be viewed. Figure 5 provides a guide explaining how to operate the “SG-eye” software while viewing the atlas (see Electronic Supplementary material for detail).

## Discussion

Degus are rodents native to the Andes Mountains in South America. In contrast to nocturnal rodents such as rats and mice, degus are diurnal rodents that can perceive information in the ultraviolet range (Chávez et al. 2003; Quirici et al. 2008). Thus, degus are useful laboratory animals for daytime behavioral experiments. Because of their intriguing social lifestyle and more advanced physical abilities than other rodents, degus are becoming more widely used in neuroscience research.

In the wild, degus live in underground burrows in small groups consisting of one or two adult males and up to five females (Ebensperger et al. 2011). Degus pups are born after a gestation period of approximately 90 days, which is much longer than the gestation period of dogs and cats (63 days). Pregnant females typically give birth to 4–8 pups at a time (range 1–12 pups) (Lee 2004). In contrast to altricial rodents, degus are born with their eyes open, a functional auditory system, teeth, and the ability to walk around the nest (Poeggel and Braun 1996; Braun and Scheich 1997; Jekl et al. 2011). Similar to human infants, degu pups are able to perceive acoustic and visual information from their social environment and can interact in rich ways with their littermates and colony mates (Colonnello et al. 2011).

The social systems of degus are unique and are quite different than the social systems of rats and mice. Degus live in tight-knit, extended family units and display complicated social behaviors, including vocal communication (Poeggel and Braun 1996). For example, lactating female degus emit characteristic nursing calls, termed mothering calls, that elicit sucking behaviors and regulate nursing bouts (Braun and Scheich 1997). These mothering calls are usually only found in mammals with strongly developed social organization, such as monkeys (Rendall et al. 2000; Owren et al. 2011), domestic pigs (Marchant et al. 2001), and rodents (Kober et al. 2008). In addition, another highly social behavior is that both the male and female degus participate in rearing their pups (Braun and Scheich 1997; Lee 2004).

Recent studies link social behaviors with brain functions. Paternal deprivation delays and partly suppresses the development of orbitofrontal neuronal circuits (Seidel et al. 2011; Braun et al. 2012). More specifically, paternal deprivation at the age of weaning (P21) results in an increase in the number of corticotrophin-releasing factor (CSF)-containing neurons in the orbitofrontal cortex and a reduction in the number of CRF neurons in the hippocampal CA1 region (Seidel et al. 2011). In addition, adult animals that were fatherless display abnormal development of layer II/III pyramidal neuron apical dendrites in the orbitofrontal cortex (Helmeke et al. 2009). This decrease may reflect decreased excitatory connectivities in this cortical subregion. Furthermore, social isolation induces a decrease in components of the limbic and monoamine neurotransmitter systems (Fuchs et al. 2010). In addition to social behaviors, brain dysfunctions related to aging can also be studied with degus. Degus would be useful for aging studies because of their much shorter lifespans than other mammals which have been often studied in this field (Inestrosa et al. 2005; van Groen et al. 2011).

However, one limitation of using degus for neuroscience research is that no detailed brain maps are available. One stereotaxic degu brain atlas is available, but this atlas only provides outlines (Wright and Kern 1992). By contrast, the atlas described in this study provides information that facilitates histological assessments of in vivo MRI data. The main advantages of this degu brain atlas include the abilities to visualize a freely movable view of the brain surface, specific brain structures in 3D, and sections in any plane (coronal, horizontal, or sagittal) with stereotaxic coordinates and structure annotations. An additional advantage of this atlas is that the stereotaxic calibrations in the maps were reconstructed by considering variations in individual animals. Thus, variations in the distance between the bregma and lambda can be used as an index to compare experimental animals with the atlas.

This atlas will also be useful for studies that assess degu brain functions with imaging modalities such as positron emission tomography (PET) and MRI. These methods have a low spatial resolution (100–500 μm). For example, in vivo MRI and PET analyses in rats have low spatial resolutions that do not permit delineation of specific brain structures (Hjornevik et al. 2007; Ravasi et al. 2011). However, in vivo imaging analyses can be combined with histological brain atlases to decipher detailed structural information. This atlas permits visualization of MR and Nissl-stained images in the same sectional plane. Therefore, when brain regions cannot be determined with MR image analyses, experimental images can be compared with the Nissl-stained images in this atlas to decipher precise brain regions. These comparisons will require appropriate adjustments of the alignment of the MR images with the atlas. To adjust the alignment, a standard zero axis (the AC–PC line, the interaural line, and the midline for the horizontal, coronal, and sagittal planes, respectively) and an outline of the MR scanned brain are the most reliable indices. In addition, the ability to perform virtual sectioning through the degu brain is a powerful function that will enable users to match their experimental images to sections with the brain atlas.

Several digital brain atlases were created from histological images (Hjornevik et al. 2007; Purger et al. 2009). For example, the BrainNavigator (http://www.brainNav.com/home/) is an interactive atlas constructed using 3D brain software. However, some of the 3D reconstructed images in the BrainNavigator atlas suffer from poor coherence of volume rendered data (Yelnik et al. 2007). For this reason, MRI-based atlases are becoming more widely used (Dorr et al. 2008). These MRI-based analyses initially utilized manual delineation (MacKenzie-Graham et al. 2004), but now utilize algorithms for semi-automated segmentation of brain structures (Ma et al. 2008; Tambalo et al. 2009; Fedorov et al. 2011; Valdés-Hernández et al. 2011; Gutierrez and Zaidi 2012). A MRI-based 3D digital atlas of the *postmortem* mouse brain is available (Lebenberg et al. 2010, 2011).

Thus, several different types of 3D digital brain atlases exist. However, this is the first 3D atlas to combine histological images with MR images and to provide users with the ability to freely move throughout the brain and cut virtual sections. Therefore, this degu brain atlas will be widely used by the neuroscience research community.

## Electronic supplementary material

Below is the link to the electronic supplementary material.

Supplementary material (pdf 126 kb)
