# Supplementary material for: Three-dimensional reconstruction of brain structures of the rodent Octodon degus: a brain atlas constructed by combining histological and magnetic resonance images
Source: Exp Brain Res. 2013 Aug 31;231(1):65–74. doi: 10.1007/s00221-013-3667-1 (PMC3824219; doi:10.1007/s00221-013-3667-1)
Supplement: Supplementary file 1 — Supplementary material (pdf 126 kb) [file 221_2013_3667_MOESM1_ESM.pdf]

The digital atlas can be operated with Windows XP, Windows Vista, Windows 7, or Mac OS X 10.5 and higher. The minimum requirements are a CPU (Core2 Duo 2.0 GHz or greater), a GPU (OpenGL compatible), more than 4 GB memory, and 20 GB of free space on the hard drive for data storage.

To download the SG-eye atlas data:

1. Go to the following URL:
   http://brainatlas.brain.riken.jp/degu/modules/xoonips/listitem.php?index_id=24
2. Download the following three files to your computer (downloading may take several minutes).
   1) Degu Brain Atlas - practical plane (sqlite) (size: 1.94 GB)
   2) Degu Brain Atlas - AC-PC conventional plane (sqlite) (size: 1.98 GB)
   3) SG-eye Viewer (size: 63.2 MB)
3. Close the URL and work with the downloaded files.
4. Open the downloaded “SG-eye Viewer” file and install by choosing the appropriate program for your operating system (SGeyeAtlas for Mac, SGeyeAtlas x32 for 32-bit Windows, or SGeyeAtlas x64 for 64-bit Windows). The folder “SG-eye v3.6” will then be installed in either your Program Files (Windows) or Applications (Mac) folder. The “SG-eye v.3.6” folder includes the “SG-eye 3D Atlas” and “SG-eye Atlas” programs.

To operate the SG-eye atlas:

(1) To operate the “SG-eye 3D Atlas” for 3D images:

1. Run the “SG-eye 3D Atlas” program to open a blank menu page.
2. Click the “Open” icon in the upper left of the program interface and then open a downloaded file (“Degu neurosurgical plane sqlite” or “Degu AC-PC radiological plane sqlite”). These files are located in the Downloads folder. The default page will then appear. This page shows a lateral view of the reconstructed degu brain (rostral and dorsal are located on the left and upper part of the reconstruction, respectively) (Fig. 5).
3. Use the mouse or the buttons on the menu bar to move the images. The functions of the buttons will appear in the tooltip window. The buttons are labeled in Fig. 5 as 1–10. There are also S, C, F, and H buttons. Several clicks of the S button will return the image to the default position.
4. Sectional views can be obtained from the reconstructed 3D atlas by manipulating the bottom right corner panel (labeled A in Fig. 5).

(2) To obtain three representative (coronal, sagittal, and horizontal) planes:

1. Click the C button several times to obtain a frontal view of the image (each click of the C button results in a rotation of 90 degrees). This will result in a series of coronal sections. Sagittal and horizontal sections can also be obtained in a similar way by using the S or H buttons. The model can be rotated by clicking the rotate button (labeled 2 in Fig. 5) or by manipulating the mouse.
2. In the bottom right corner panel (labeled A in Fig. 5), click the boxes labeled “C” and “Move with” in the “Clipping” and “Plane” modes in the “Axial” tab. These modes can be activated by clicking the tags with their names. Move the “C” slider with the mouse to change levels along the rostro-caudal axis. The numbers indicated in the boxes to the right of the sliders are the distance in mm from the interaural zero line. These numbers are either positive or negative to signify the rostral and caudal positions relative to the zero line, respectively. Nissl and outline images can also be observed at all of the rostro-caudal levels. 3D image or outlines can be removed or added by clicking the appropriate buttons (labeled 6 and 7 in Fig. 5). MR images can also be added by clicking the appropriate button (labeled 8 in Fig. 5).
3. Nissl sections, 3D images, MRI planes, and outlines can be visualized in any combination in the “Free” tab in the bottom right corner panel (labeled A in Fig. 5). This requires clicking the appropriate buttons (labeled 2, 6, 7, and 8 in Fig. 5) and clicking the boxes labeled “C” and “Move with” to move the sliders (C, S, H, or various combinations) in the appropriate direction. Click the appropriate button (labeled 9 in Fig. 5) to reset the image.
4. Hierarchical brain structure compositions are indicated in the middle of the right panel (labeled B in Fig. 5). Representative structures with unique colors are shown in the outlines.
5. Stereotaxic coordinates can be obtained by using the appropriate button (labeled 10 in Fig. 5). The images can be freely moved with the rotate button (labeled 2 in Fig. 5) and sections can be visualized in the “Axial” or “Free” tabs of the bottom right control panel (labeled A in Fig. 5).
6. To change files, click “File” and then “Open” to choose a different data set.

(3) To operate the “SG-eye Atlas” for 2D images:

1. Run the “SG-eye Atlas” program to open a blank menu page.
2. Click the “Open” icon in the upper left of the program interface and open a downloaded file (“Degu neurosurgical plane sqlite” or “Degu radiological AC-PC plane sqlite”). These files are located in the Download folder. The default page will then appear. This page shows a lateral view of the reconstructed degu brain (rostral and dorsal are located on the left and upper part of the reconstruction, respectively). The Nissl images in the neurosurgical plane were acquired from a series of coronal sections, and the images in the radiological AC-PC coronal plane were reconstructed from the 3D model.
3. Familiarize yourself with the functions of the active buttons (the functions will appear in the tooltip window and are described above). The essential functions include selection of brain areas,

observing the background image, addition of MR images, displaying structures demarcated with unique colors, drawing grid lines, and indicating stereotaxic coordinates. The magnification of the images can be changed by moving the slider in the upper left corner of the main panel.

4. The panels in the menu page are: lateral view of a reconstructed degu brain (upper left), which shows images at the level of individual sections; a series of sections with stereotaxic scales (upper middle); a navigator panel (upper right); and a list of color coded brain areas arranged structurally in a hierarchical manner (middle right).

Images can be loaded in the central panel (click the “background” button and then click one of the serial sections). MR images can be added by clicking the upper right corner (the small “MRI button”). Brain areas can be indicated in the loaded image by clicking the appropriate button (the “show structure” button). The cursor can moved to any brain region by clicking the appropriate button (the “selection” button). In addition, a left click of the mouse will annotate the structural hierarchy of the selected areas. Stereotaxic coordinates can be acquired by clicking the appropriate buttons (the “grid” and “scale” buttons).
